# Effect of the Degree of Crystallinity of Base Material and Welded Material on the Mechanical Property of Ultrasonically Welded CF/PA6 Joints

**DOI:** 10.3390/ma18020420

**Published:** 2025-01-17

**Authors:** Ruoya Shi, Mingyang Li, Sansan Ao, Yang Li

**Affiliations:** School of Materials Science and Engineering, Tianjin University, Tianjin 300354, China; sry197801@tju.edu.cn (R.S.); lmytju2022@163.com (M.L.); ao33@tju.edu.cn (S.A.)

**Keywords:** CF/PA6, degree of crystallinity (DoC), ultrasonic welding, heat treatment

## Abstract

Ultrasonic welding (USW) is considered one of the most suitable methods to join semi-crystalline carbon fiber-reinforced thermoplastics (CFRTPs). The degree of crystallinity (DoC) of the semi-crystalline resin will affect the ultrasonic welding process by affecting the mechanical properties of the base material. In addition, ultrasonic welding parameters will affect the joint performance by affecting the DoC of the welded material at the welding interface. This paper investigates the effect of DoC of carbon fiber-reinforced PA6 (CF/PA6) base material and welded material on its ultrasonically welded joints’ performance. Distinct pre-welding heat treatments are conducted on the base material before welding. The DoC is calculated by DSC, while the crystalline phases (α and γ phases) and crystallize size are determined using XRD. The results demonstrate that the heat treatment process of heating temperature of 180 °C and cooling with an oven (180-O) could increase the DoC of CF/PA6 from 27.2% to 33% and the ratio of α/γ from 0.38 to 0.75. The joint strength of 180-O sheets reached 17.7 MPa, which is 26.7% higher than that of the as-received sheets. The DoC of the welded material at the welding interface obtained with different combinations of welding parameters is characterized. Higher welding force and amplitude result in faster cooling rate at the welding interface and more effective strain-induced crystallization, leading to higher DoC of the welding interface.

## 1. Introduction

As a successful commercial engineering polymer, polyamide 6 (PA6) has been widely used in the automotive, electronics, construction, and packaging fields, etc. [1,2], due to its excellent physical and mechanical properties [3,4]. Currently, in addition to the standalone application of PA6, its use as a matrix for carbon fiber-reinforced thermoplastic composites (CFRTPs) is expanding [5,6]. The application of CFRTPs in the construction of structural components has fostered extensive research into their suitable joining processes, given the necessity for the fabrication of substructures and their subsequent assembly. The property of repeated melting and cooling of the thermoplastic matrix makes the welding methods a viable option for joining CFRTPs. A synthesis of multiple evaluation criteria indicates that ultrasonic welding (USW) is a prospective technology for industrial manufacturing applications, characterized by energy efficiency, simple operation, cost-effectiveness, and in situ monitoring capability [7,8].

So far, most efforts in the fields of CFRTP USW have focused on the study of the heating and healing mechanisms of the material, as well as the potential for enhancing the joint quality through the optimization of welding process parameters and the form of energy directors [4,9,10,11,12,13]. However, in the case of semi-crystalline polymers such as PEEK, PPS, PA6, and so on, the mechanical properties are dependent, to some extent, on the degree of crystallinity (DoC). The aforementioned mechanical properties, which include stiffness, tensile strength, ductility, and toughness, subsequently influence the joint quality [14,15]. Taketa et al. [16] examined the DoC and static bending properties of CF/PA6 acquired at varying cooling rates, and the findings indicated that as the cooling rate increased, the crystallinity declined, accompanied by a reduction in matrix modulus. Previous research has also revealed a positive correlation between DoC and the tensile properties of PEEK [14]. The high modulus of elasticity and low damping coefficient of CFRTP are indicative of its favorable acoustic properties, which facilitate the transmission of ultrasonic vibrations at the welding interface [8,17]. Conversely, experiments conducted with carbon experiments conducted with carbon fiber-reinforced PAEK have demonstrated that an increase in DoC is associated with a reduction in interlaminar fracture toughness [15]. Additionally, the annealing process has been observed to enhance the uniformity of the crystallinity distribution of carbon fiber-reinforced PA6 (CF/PA6) workpieces and mitigate the uneven distribution of the material morphology present during injection molding [18]. The CFRTP USW heat-generation mechanism demonstrates that frictional and viscoelastic heat generation exerts a predominant role across distinct phases of the welding process. The storage modulus and loss modulus, which are dependent on the material’s crystallinity, affect two forms of heat generation that occur during the USW process. Consequently, these properties affect the joint quality. Therefore, the DoC of the base material is recognized as a factor affecting the weld performance. Nevertheless, there is a lack of research focus on the effect of DoC on weld quality.

In addition to the DoC of the base material, the ultrasonic welding parameters result in the material at the welding interface undergoing a distinct thermal cycle to that of the base material, thereby impacting the DoC of the welded material. Generally, the ultrasonic welding process is characterized by remarkably short times, which results in a correspondingly fast cooling rate at the interface. The welding process parameters could affect the cooling rate, which in turn affects the DoC of the welded material [10,18]. This will, theoretically, influence the mechanical properties of the welded joint. However, the current research is also limited in this domain; only Koutras et al. [19] investigated the effect of welding force and amplitude process parameters in combination on the DoC of welded joint of CF/PPS.

This paper characterizes the DoC, the mass fraction of the two crystalline forms (α and γ) and the crystal size of CF/PA6 subjected to different pre-welding heat treatments. Additionally, it elucidates that the strength of ultrasonically welded joints is influenced by heat treatments, which modify these intrinsic characteristics of the material, since the factors affecting the mechanical properties of the joint include not only the energy transferred to the interface, but also the physical state of the material at the interface. This paper characterizes the cooling rate, DoC and weld area at the interface for different combinations of welding force and amplitude. Furthermore, it investigates the relationship between the process parameters and joint performance from the perspective of the DoC.

## 2. Materials and Methods

### 2.1. Material and Experimental Set-Up

**Figure 1:**
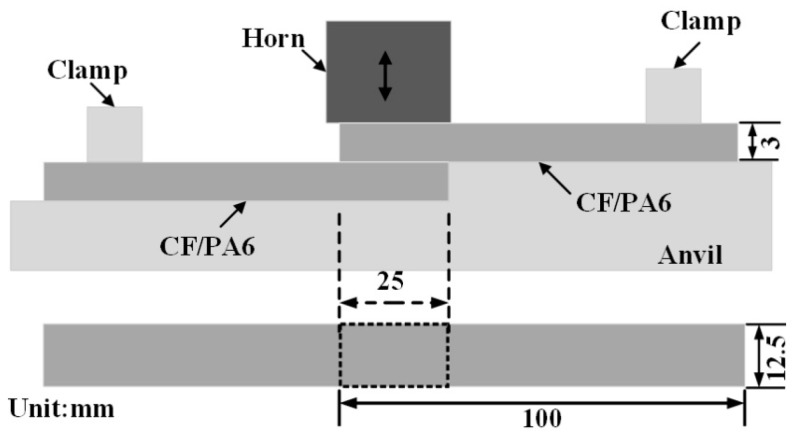
The experimental set-up.

Short carbon fiber-reinforced polyamide 6 (CF/PA6) containing 30wt% carbon fiber was employed in this research. The dimensions of all sheets were 100 × 25 × 3 mm^3^, and the molding process employed was injection molding. The welded joints were made using a 20 kHz ultrasonic welder (Herrmann VE20 SL DIALOG 6200, Herrmann Ultrasonics (Taicang) Co., Ltd., Taicang, China). Figure 1 depicts the experimental set-up. A rectangular steel horn with dimensions of 20 mm × 30 mm was utilized. The workpieces were subjected to welding, with an overlapping area of 12.5 × 25 mm^2^.

The experiments in this study were divided into two parts. The first part investigated the impact of the DoC of the base material on the characteristics of the ultrasonically welded joints. A number of CF/PA6 sheets with varying DoC were obtained through heat treatment prior to welding. In this section, energy directors (EDs) were not used for welding, in order to avoid any potential effect on DoC. Table 1 lists the welding parameters. Five replicas were conducted for each group of welding parameters.

The second part characterized the DoC at the interface and joint strengths for different combinations of welding parameters. Based on the optimum pre-weld heat-treatment process determined in the first part, the welding parameters shown in Table 2 were used to manufacture joints with different DoCs. Similarly, five replicas were conducted for each group of welding parameters. In contrast to the first part, to avoid random welds and to facilitate the removal of material from the welding interface, flat EDs were used. All flat EDs were dried at 80 °C for 4 h, prior to welding. This not only removed moisture, but also ensured that the EDs exhibited approximately the same initial DoC.

### 2.2. Heat-Treatment Process

**Figure 2:**
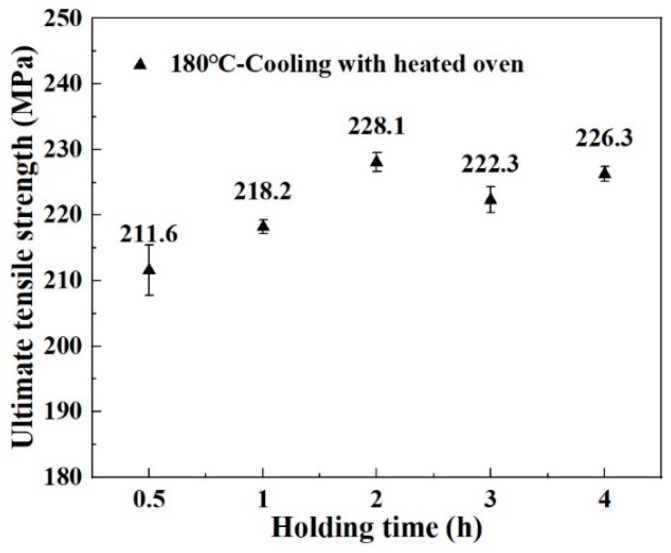
Ultimate tensile strength of CF/PA6 obtained after holding at 180 °C for different times.

**Figure 3:**
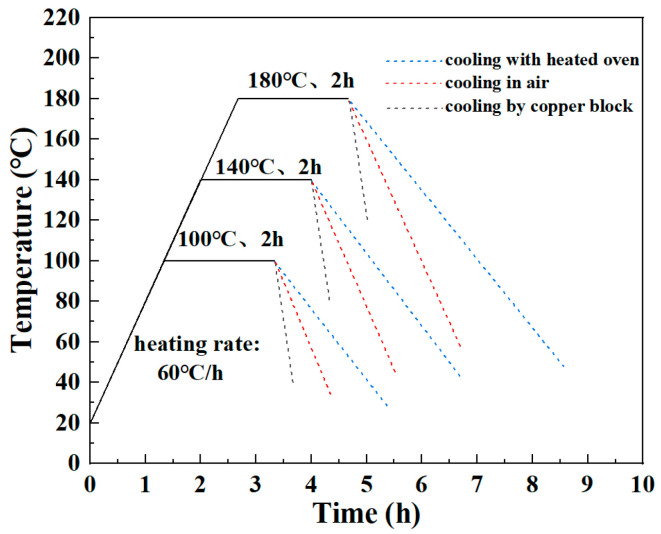
The heat-treatment curve.

Heat treatment was used to obtain CF/PA6 sheets with different DoCs before welding. The process parameters included heating and cooling rates, heat-treatment temperature, and holding time. In the context of polymer treatment, it is typically necessary to utilize a temperature above the Tg of the material, while remaining below its melting point (Tm). The heating rate is maintained below 120 °C/h [18]. In this study, different heat-treatment temperatures (100, 140, and 180 °C) were used, and to avoid warm flushes during the heating process and to reduce time costs, the heating rate was selected as 60 °C/h. In addition, different cooling rates were achieved by using different cooling modes (cooling with a heated oven, cooling in air, and cooling by a copper block). Between the heating and cooling phases, the holding time was also an important factor. Preliminary experiments were conducted to determine the optimum holding time. Five samples were tested for each holding time. Figure 2 shows the ultimate tensile strength (UTS) of CF/PA6 sheets obtained at 180 °C for different holding times. The results show that the UTS initially increased in conjunction with an elongation of holding time. However, it decreased slightly when the time exceeded 2 h. The diffusion of polymer chains is time-dependent, so an increase in holding time favors the rearrangement of more molecular chain ends to achieve an efficient and regular arrangement [20]. As the holding time increased, the DoC gradually increased, increasing the UTS. Nevertheless, the advancement of crystallinity ultimately impedes the mobility of molecular chain segments in both the amorphous and crystalline regions, thereby hindering further enhancements in the material’s crystallinity and ultimate tensile strength (UTS) [21]. Therefore, 2 h was chosen as the holding time in this study. Workpieces in the absence of any processing are designated as as-received. The heat-treatment curves are shown in Figure 3. Table 3 lists the heat-treatment processes and their abbreviations. Five samples were tested for each heat-treatment process.

### 2.3. DSC Analysis

**Figure 4:**
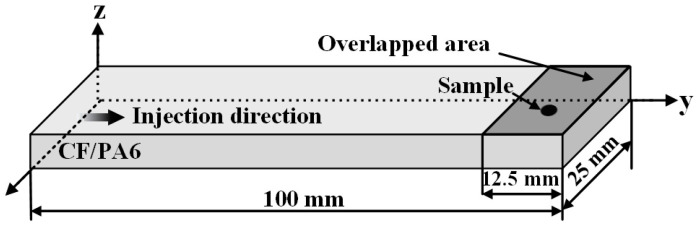
The sampling position and location for the DSC tests.

For heat-treated and as-received CF/PA6 sheets, DSC samples were collected from the overlapped area to be welded away from the injection molding end, as illustrated in Figure 4. The samples obtained from the joints made with the welding parameters shown in Table 2 are denoted by ED_HF-HA, ED_MF-MA, and ED_LF-LA, respectively. The ED film in the initial state (after drying but prior to welding) is denoted by ED_ref.

The DSC tests were conducted using a NETZSCH DSC 214 Polyma machine (Selb, Germany). Specimens of approximately 5 mg were sealed in aluminum pans. All tests were conducted in a nitrogen atmosphere (set to 40 mL/min). The specimens were treated with a heating process, increasing the temperature from room temperature to 280 °C at 10 °C/min, to record their melting behavior. The DoC was calculated using Equation (1) [22]:

(1) $$DoC=\frac{\Delta H_{m}-\Delta H_{cc}}{\alpha \times \Delta H_{f}^{0}}\times 100\%$$

where α is the weight fraction of the matrix (70%), $\Delta H_{m}$ and $\Delta H_{f}^{o}$ represent the melting enthalpies of the calculated and 100%-crystalline PA6, respectively ($\Delta H_{f}^{o}$ = 240 J/g).

### 2.4. XRD Analysis

XRD measurements were made using a D8 ADVANCE X-ray diffractometer. The scanning speed was set to 0.02 °/s, with the diffraction angle (2θ) ranging from 10° to 30°. The experimental curves were fitted to the peak functions based on the raw XRD mapping data using Fityk software (v4.12). The appearance of the α form in PA6 is usually accompanied by two characteristic peaks, α1 (2θ ≈ 20°) and α2 (23.7°) [23], while the γ is characterized by a strong peak at 21.3° (γ1) and a weak peak at 22° (γ2) [24]. Furthermore, amorphous PA6 and carbon fibers display broad diffraction peaks at approximately 2θ ≈ 21.4° and 2θ ≈ 25.5° [25], respectively. The fitting calculations for each peak curve were performed using the Gauss–Voigt shape model, and the height and width of each peak were manually adjusted before starting the automatic fitting, to provide a reasonable starting point. The fit coefficients for each curve were as high as 0.99, indicating that the model predictions were highly aligned with the experimental data. To quantify the fractions of the α and γ forms (X_α_, X_γ_), as well as the ratio of α/γ, the equations given below were used [19].

(2) $$X_{\alpha }=\frac{A_{\alpha 1}+A_{\alpha 2}}{A_{\alpha 1}+A_{\alpha 2}+A_{\gamma 1}+A_{\gamma 2}+A_{\mathrm{amorphous}}+A_{CF}}$$

(3) $$X_{\gamma }=\frac{A_{\gamma 1}+A_{\gamma 2}}{A_{\alpha 1}+A_{\alpha 2}+A_{\gamma 1}+A_{\gamma 2}+A_{\mathrm{amorphous}}+A_{CF}}$$

(4) $$\frac{\alpha }{\gamma }=\frac{X_{\alpha }}{X_{\gamma }}$$

The α and γ forms of PA6 have different structural parameters, as shown in Table 4.

The average crystalline size was calculated by the following equation [26]:

(5) $$D=K\lambda /\left(\beta \cos\theta \right)\left(\overset{{}^{\circ}}{A}\right)$$

where *θ* is the Bragg angle, *K* represents the shape factor (0.94), *λ* denotes the X-ray wavelength (1.54 Å), and *β* is the half-height width of the diffraction peak.

### 2.5. Temperature Measurement

The temperature at the edge of the overlapping area was measured, due to the obvious edge effect present in this study; 0.1 mm diameter K-type thermocouples were affixed manually to the PA6 films, using an adhesive type. A sampling frequency of 100 Hz was employed for the temperature data, utilizing an Arduino development board.

## 3. Results

### 3.1. Effect of the DoC of CF/PA6 Base Material on the USW Process and Joint Strength

#### 3.1.1. Effect of Heat Treatment on the DoC of CF/PA6

**Figure 5:**
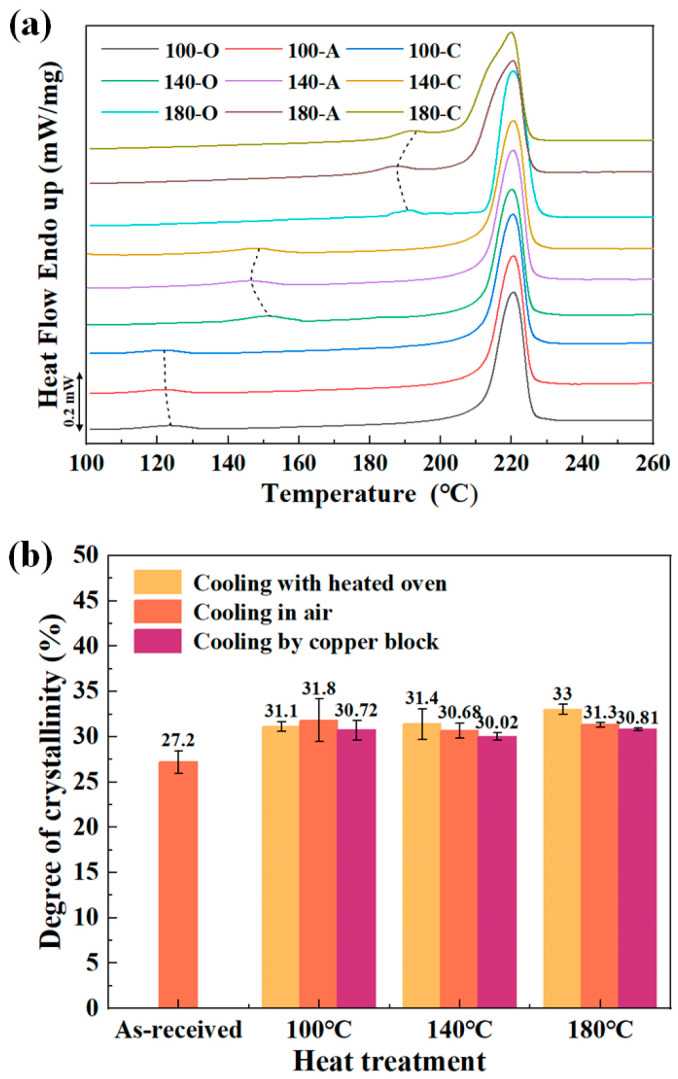
The DSC results: (**a**) the DSC curves; (**b**) the DoC results.

Figure 5 illustrates the DSC heating curves of CF/PA6 and DoC results obtained after the heat-treatment process. Two distinct endothermic peaks were observed above Tg: the classical melting, located at a higher temperature (II), and the other, a smaller peak, reflected at a lower temperature (I). This phenomenon has also been observed in other materials [27,28]. Accordingly, the temperatures of the two peaks are denoted as lower peak temperature T_m1_ and higher peak temperature T_m2_. As the heat-treatment temperature increased, T_m1_ exhibited a shift towards higher temperatures. It was observed at 120 ± 2 °C for heat treatment at 100 °C and increased up to 190 ± 2 °C for heat treatment at 180 °C. For various curves, T_m1_ was found to occur 10~20 °C above the heat-treatment temperature, while T_m2_ remained constant, as shown in Table 5. In addition, the size of the small endotherms was observed to increase in conjunction with the heat-treatment temperature.

Given that the melting point of polymers is proportional to the lamellar thickness [29], the occurrence of multiple melting behaviors on the DSC heating curves can be explained by the heterogeneous distribution of lamellar thickness within the CF/PA6 material following heat treatment. The differing abilities of molecular chains to move result in a range of lamellar thickness within the original crystal structure of the material, which encompasses both thin and thick crystals. When CF/PA6 is subjected to heat treatment at temperatures above the T_g_, a portion of the thin lamellae melts, due to its low melting point. Furthermore, the external heat source provides the energy necessary for the movement of the molecular chains, which then reorganize into new crystals or re-enter the crystalline layer to undergo re-crystallization. This results in a thickening of the lamellae. The thin lamellae are prone to undergoing a process of organization that results in the formation of more stable and thicker lamellae. Consequently, primary thick and secondary crystalline crystals will display disparate melting behaviors, resulting in the occurrence of multiple melting phenomena.

The melting enthalpy of specimens was calculated by summing the areas under the two endothermic peaks, as determined by Equation (1), to ascertain the DoC. Figure 5b shows the DoC of CF/PA6 sheets obtained under different heat treatments. The DoC of the as-received CF/PA6 was only 27.2 ± 1.2%. After heat treatment at 100, 140 and 180 °C, the maximum average DoC reached 31.8 ± 2.4%, 31.4 ± 1.7%, and 33 ± 0.6%, respectively. It shows that the DoC increased after the heat-treatment process. This is because the polymer molecular chains are activated at high temperatures, and the soft segments of the molecular chains move and become loose, which makes the original molecular chains with defects enter the crystal lattice, after obtaining energy, rearrange into an ordered structure, leading to an increase in DoC.

The maximum difference in DoC among the three cooling modes at 100, 140 and 180 °C were 0.4%, 1.4% and 2.2%, respectively, which was almost negligible, indicating that the cooling phase of the heat-treatment process did not affect the crystallization.

#### 3.1.2. Effect of Heat Treatment on the α/γ Ratio and Crystalline Size

**Figure 6:**
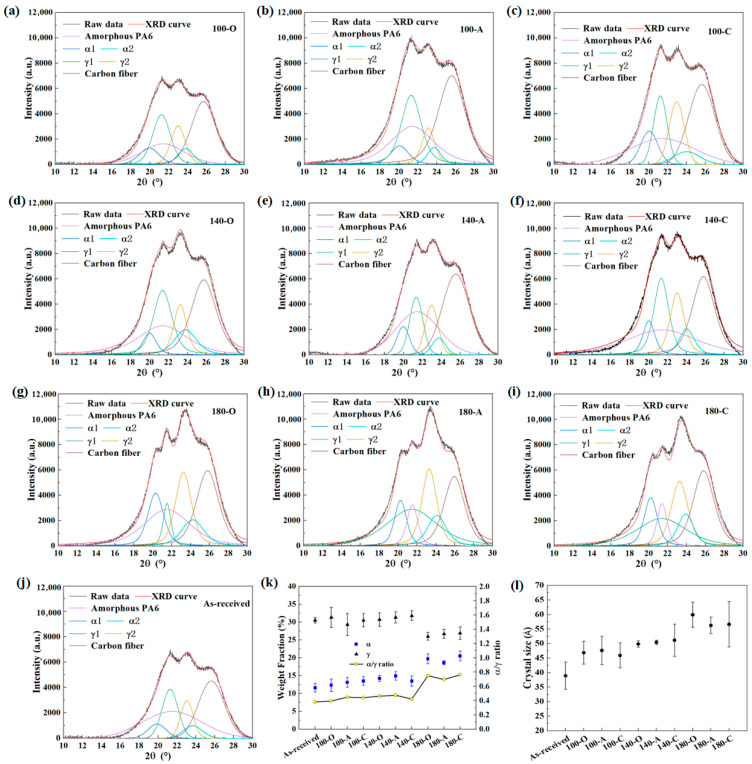
The XRD results of samples obtained with different heat treatments: (**a**–**c**) 100 °C; (**d**–**f**) 140 °C; (**g**–**i**) 180 °C; (**j**) as-received; (**k**) the fractions of the α and γ phases and the α/γ ratio; (**l**) crystal size.

Figure 6 illustrates the XRD original and fitted-curve plots. Compared with the as-received, the shape and intensity of the diffraction patterns of the heat-treated workpieces changed significantly, especially at 180 °C. This indicates that the pre-welding heat treatment had an impact on the crystal structure. The specific values of the proportions of each crystal form are depicted in Figure 6k. The results show that the proportion of the γ phase was significantly higher than that of the α phase. This is because the cooling rate at the surface of the injection-molded workpiece is faster, which is conducive to the formation of the γ form. At 100 and 140 °C, the α and γ forms both exhibited a slight increase, while the α/γ ratio remained relatively constant. This is because the amorphous phase can be transformed into both crystal forms within this temperature range, with minimal transformation between the two crystal forms. Conversely, at 180 °C, a considerable increase in the α form and a significant decrease in the γ form were observed, indicating that at this temperature, not only was the amorphous phase transformed into the α form, but the γ form was also transformed into the α form. Additionally, the impact of the cooling mode on the crystalline-phase composition of the CF/PA6 was practically negligible.

The average crystalline size of as-received CF/PA6 was 38.9 ± 4.7 Ả. When the pre-welding heat treatment was used, the maximum values at 100, 140 and 180 °C were 47.6 ± 4.8 Ả, 51.1 ± 5.5 Ả and 59.9 ± 4.4 Ả, respectively. It was consistent with the pattern of the effect of heat treatment on DoC, i.e., promoting the growth of the crystalline regions. This is because the mobility of the PA6 molecular chains is enhanced as a consequence of the elevated temperature, resulting in sufficient activation for the migration and rearrangement of the molecular chains, which promotes crystal growth and effectively improves the integrity of the crystals [28,30]. Compared with the temperature, the effect of the cooling mode was very slight, and there was no regular pattern, indicating that the crystal growth mainly occurred during the holding phase of the heat-treatment process.

#### 3.1.3. Effect of Heat Treatment on the Joint Strength

**Figure 7:**
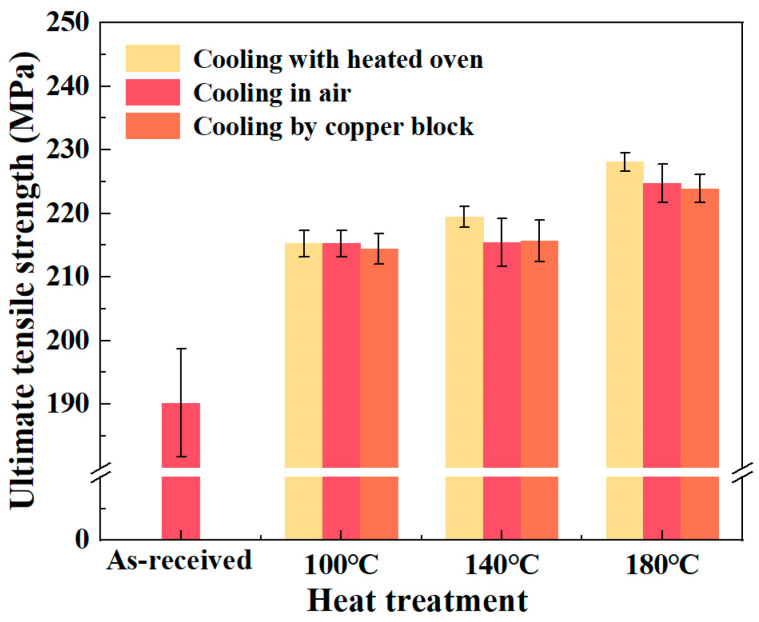
The UTS of CF/PA6 under different heat-treatment processes.

The effect of the heat-treatment process on the UTS of CF/PA6 is shown in Figure 7. It shows that the UTS increased with the increasing heat-treatment temperature. As the DoC increases, the molecular chains become more compact and ordered, and the intermolecular interaction pressures increase, which impedes the movement of the chain ends and improves the UTS of CF/PA6 after heat treatment. The results in Figure 7 show that the cooling mode affected the tensile strength of the material in a more obvious pattern, i.e., the CF/PA6 sheets cooled with a heated oven had the highest UTS, while those cooled by a copper block had the lowest UTS. On one hand, this can be attributed to the variation in the DoC of the material; on the other hand, cooling in air and cooling by copper block create a greater cooling-rate difference between the material surface and the interior of the material, which in turn causes internal stress in the material and reduces UTS.

**Figure 8:**
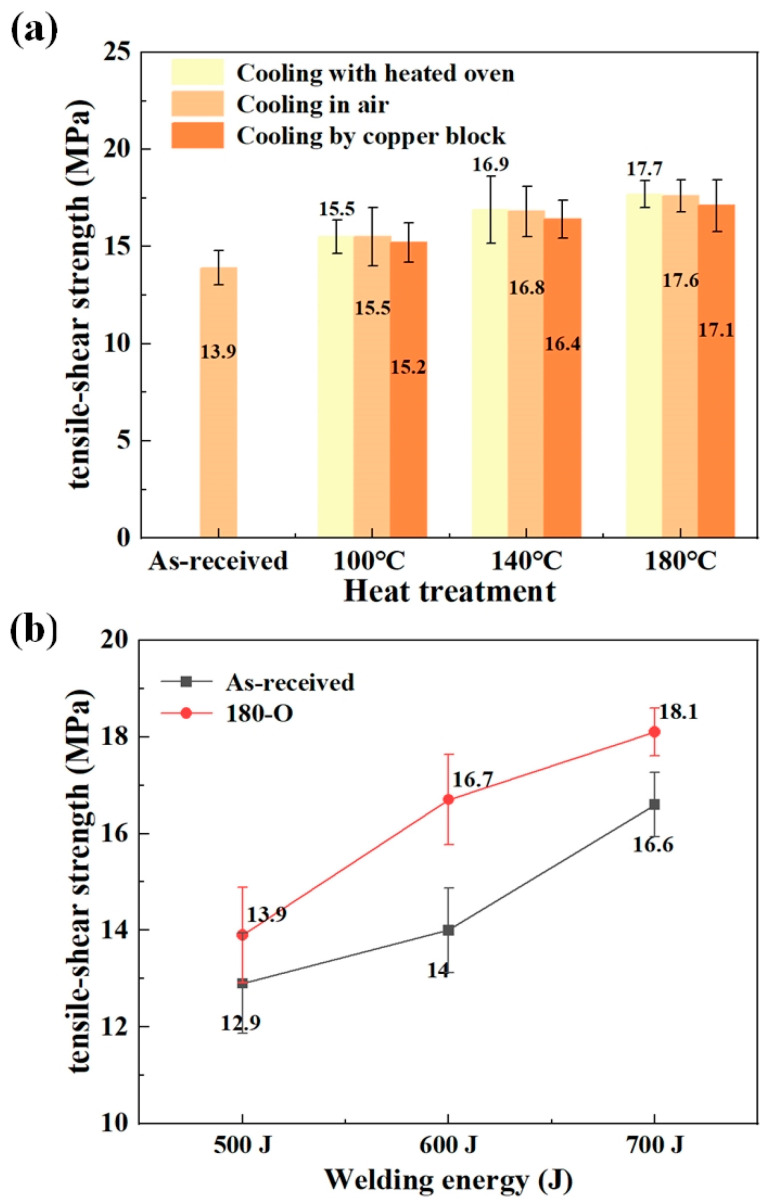
Tensile–shear strengths of joints (**a**) made with as-received and heat-treated CF/PA6 sheets; (**b**) made at different welding energies (500, 600, and 700 J).

Figure 8 presents the tensile–shear strength of joints. In the case of using as-received CF/PA6 sheets, the joint strength was 13.95 ± 0.87 MPa. If the pre-welding heat treatment was conducted, the maximum joint strengths obtained at 100, 140 and 180 °C were 15.5 ± 1.5 MPa, 16.8 ± 1.3 MPa and 17.7 ± 0.7 MPa, respectively. The maximum difference in joint strengths among the three cooling modes at 100, 140 and 180 °C were 1.9%, 2.8% and 3.5%, respectively. Therefore, the joint strength can be enhanced by pre-welding heat treatment of base material. In addition, the joint strength tended to increase with increasing heat-treatment temperature, whereas there was almost no difference in joint strength among the three cooling methods. These results proved the influence of DoC and the crystalline structure of CF/PA6 sheets on the USW process. It is believed that higher DoC and α crystalline form contribute to higher modulus, which in turn leads to increasing interfacial frictional heat generation, and therefore, improved weld quality.

For 180-O, different welding energy levels were adopted to verify the positive effect of heat treatment on the USW process. The joint strengths at welding energy of 500 J for as-received and 180-O were 12.9 ± 1.1 MPa and 13.9 ± 1.0 MPa, respectively. When raising the welding energy to 700 J, they increased, respectively, to 16.55 ± 0.66 MPa and 18.1 ± 0.5 MPa. The joint strength can be enhanced at various welding energies by applying the pre-welding heat treatment. This proved that the higher DoC and α crystalline form was associated with better weld performance.

**Figure 9:**
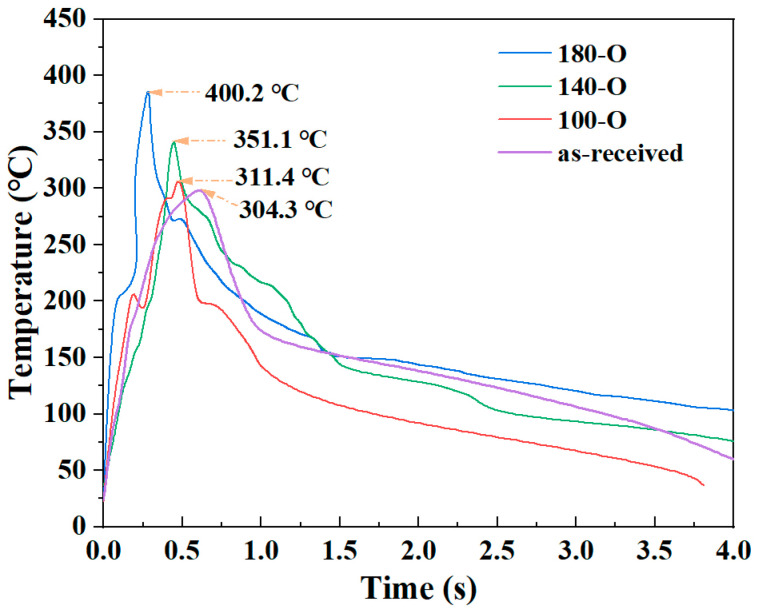
The temperature evolution during the USW process.

Figure 9 depicts the temperature evolution during the USW process. It revealed a notable difference in the temperature evolution of the USW process between as-received and heat-treated CF/PA6 sheets. Compared to the as-received, the pre-welding heat treatment resulted in a significant acceleration in the rate of temperature and maximum temperature. In addition, 180-O led to the fastest heating rate and highest maximum temperature. This is further evidence that the increased DoC and α/γ ratio improves the mechanical properties of the CF/PA6 base material, leading to higher interfacial heat generation.

### 3.2. Effect of Welding Parameters on the DoC at the Welding Interface and Joint Strentgth

#### 3.2.1. Effect of Welding Parameters on the Cooling Rate at the Welding Interface

**Figure 10:**
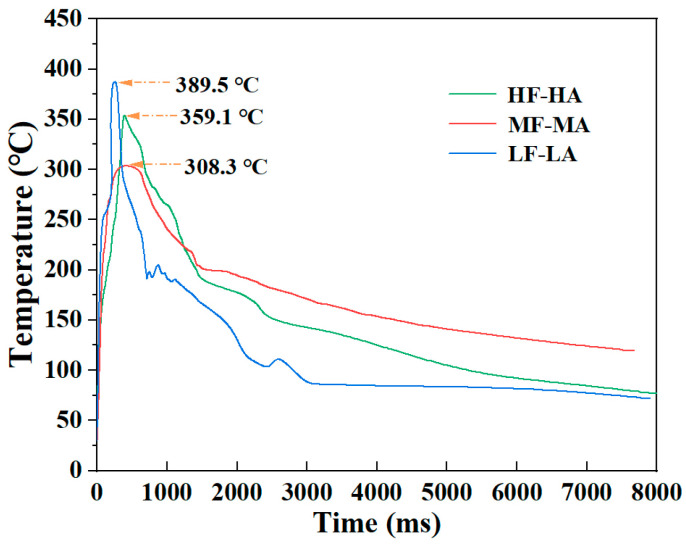
Temperature evolution during ultrasonic welding of HF-HA, MF-MA and LF-LA.

It is evident that variations in welding force and amplitude exert a considerable effect on the power dissipated and vibration time during ultrasonic welding processes [31]. As shown in Table 6, an increase in welding force and amplitude was observed to result in higher dissipated power and faster heating rates, thereby shortening the vibration times for specified welding energy. Figure 10 illustrates the temperature evolution for a single specimen under each experimental condition. The relationship between time and temperature was non-linear, which means that it is not possible to calculate the global cooling rate with accuracy. Alternatively, the temperature between the melting point of CF/PA6 (221~223 °C) and 100 °C was segmented into two distinct temperature regions, where the relationship between time and temperature was approximated as linear. The results are shown in Table 6. Due to the deceleration of thermal convention with decreasing temperature, the cooling rates of all specimens decreased with time. The cooling rates of HF-HA, MF-MA and LF-LA between 100 °C and 150 °C were 58.4 °C/s, 49.1 °C/s and 18.4 °C/s, respectively. As illustrated in Figure 10 and Table 7, an increase in both welding force and amplitude has been observed to result in notable enhancement in cooling rates. This is mainly because when using HF-HA, the welding time was shorter, the heat generated by welding had not yet significantly heated the surrounding workpieces, the temperature difference between the welding zone and the unwelded area was higher, and the cooling rate was fast. Conversely, the use of LF-LA prolonged the welding time, resulting in the surrounding workpieces being heated to a higher temperature. This, in turn, led to lower temperature in the welding area, with a consequent reduction in the temperature differential between the welding zone and the unwelded area, and a slower cooling rate.

#### 3.2.2. Effect of Welding Parameters on the DoC at the Welding Interface

**Figure 11:**
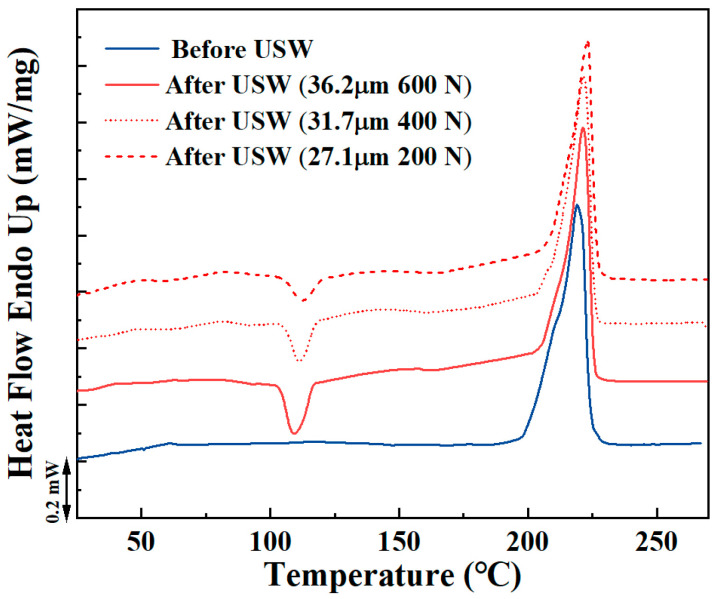
Heating curves of DSC measurements.

Figure 11 illustrates heating curves observed during the DSC measurements carried out on ED_ref and specimens at the welding interface. The ED_ref did not display an exothermic peak, whereas the remaining three specimens exhibited exothermic peaks at approximately 190 °C, due to cold crystallization. The phenomenon of cold crystallization occurs above the Tg during the heating process from the glassy state. This indicates that partial crystallization occurred during the preceding cooling period, due to the cooling rate being too fast. The enthalpy correlated with cold crystallization is proportional to the PA6 fraction that did not have sufficient time to undergo full crystallization during cooling before the DSC heating. The most pronounced exothermic peak was observed in the ED_HF-HA specimen, indicating that the high welding force and amplitude resulted in more molecular chains being left to crystallize during the cooling process. Furthermore, one endothermic peak corresponding to the melting of PA6 around 222 °C (±1 °C) was observed per specimen. The average DoC calculated by Equation (1) is presented in Table 8. The DoC of ED_ref was 31.1%. Concerning ED_ref, ultrasonic welding facilitated the production of ED films characterized by moderate crystallinity. It is also worth noting that the considerable scatter of DoC values was observed in ED films obtained at the welding interfaces, as presented in Table 8. The considerable experimental error observed in the DoC values may be attributed to the non-uniform crystallization during cooling.

It is well known that a faster cooling rate leads to lower DoC [32]. When the cooling rate exceeds 100 °C/s, PA6 is no longer crystalline, and is mainly amorphous [33]. As shown in Table 7, the cooling rates in the range of 150~220 °C were around 100 °C/s or higher. This indicates that the crystallization of PA6 in the welding process occurred mainly in the temperature range of 100~150 °C. In this temperature region, lowering the welding force and amplitude resulted in a significant decrease in the cooling rate, facilitating adequate time for molecular chain recombination. In this case, ED_LF-LA should exhibit a higher DoC; however, the results in Table 8 are inconsistent with this conclusion. This is because the factors that affect the crystallization of a material include not only temperature, but also force.

One significant distinction between the crystallization of PA6 in ref. [32] and during ultrasonic welding is that the former occurs in a static condition, whereas the latter is subjected to a high stress and strain rate. The vibration frequency employed in this study for ultrasonic welding was 20 kHz, thereby enabling the attainment of exceedingly high strain rates, instantaneously. In a static condition, the crystallization is solely dependent on the mobility of the molecular chains and the supercooling level. If the cooling kinetics exceed those of crystallization, the crystallization behavior will be prevented, resulting in amorphous PA6. Nevertheless, when it comes to strain, it can potentially affect DoC through different mechanisms. It is well known that high strain rates have a considerable impact on the crystallization kinetics of polymers, given that the polymer molecular chains are highly oriented under force conditions [34]. The strain-induced orientation of these molecular chains can cause notable changes in the nucleation and crystal growth rates of the polymers, which in turn promotes crystallization behavior [19,34]. Higher welding force and vibration amplitude can create more favorable conditions for nucleation, leading to the growth of more crystalline regions. Therefore, it is proposed that the crystallization behavior of PA6 exposed to extremely rapid cooling rates can be explained by the mechanism of strain-induced crystallization. An increase in welding force and amplitude resulted in an elevated cooling rate at the interface, which was detrimental to the crystallization. Conversely, this process could also facilitate the alignment of molecular chains and accelerate the crystallization, thereby enhancing the DoC. These two opposing effects compete for dominance in determining the final DoC at the interface. In conclusion, the DoC at the welding interface is attributed to the heat generation and force during the welding process. The results demonstrate that DoC at the ultrasonically welded interface may be controlled through applied force and amplitude.

#### 3.2.3. Effect of Welding Parameters on Joint Strength

**Figure 12:**
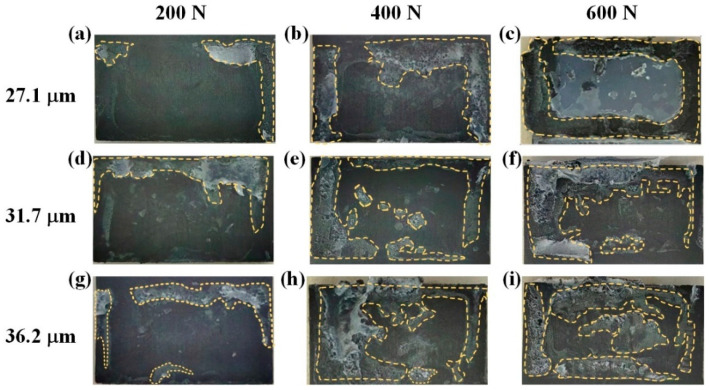
The shape of the melt region of ultrasonically welded CF/PA6 joints under different welding parameters: (**a**) 200 N/27.1 μm; (**b**) 400 N/27.1 μm; (**c**) 600 N/27.1 μm; (**d**) 200 N/31.7 μm; (**e**) 400 N/31.7 μm; (**f**) 600 N/31.7 μm; (**g**) 200 N/36.2 μm; (**h**) 400 N/36.2 μm; (**i**) 600 N/36.2 μm.

**Figure 13:**
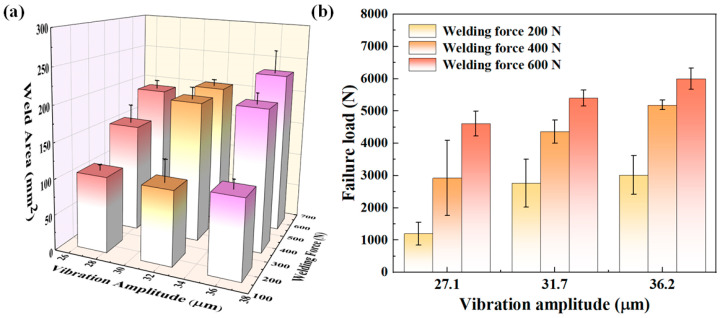
The tensile–shear test results of CF/PA6 joints: (**a**) weld area; (**b**) joint failure loads.

Figure 12 shows the melting regions of CF/PA6 joints, with the weld contour highlighted by yellow dashed lines. When the welding force or amplitude was low, a distinct edge effect was exhibited at the weld, i.e., only a small area at the edge region melted, as shown in Figure 12a,d,g. This is because the stress concentration occurred at the edge area of the flat ED. As the welding force and vibration amplitude increased, welds gradually grew toward the center of the overlap area and the measured weld area became larger. This agrees with the measurements in Figure 13a. In addition, it can be seen from Figure 13a that the amplitude has limited influence on the weld area, while the welding force has significant effect on the weld area. This is because when the welding force was low, the initial contact area between the workpieces was smaller, and the welding energy can only act on a smaller amount of welding material. At this point, the change in amplitude cannot change the contact area, resulting in a smaller impact of amplitude on the weld area.

**Figure 14:**
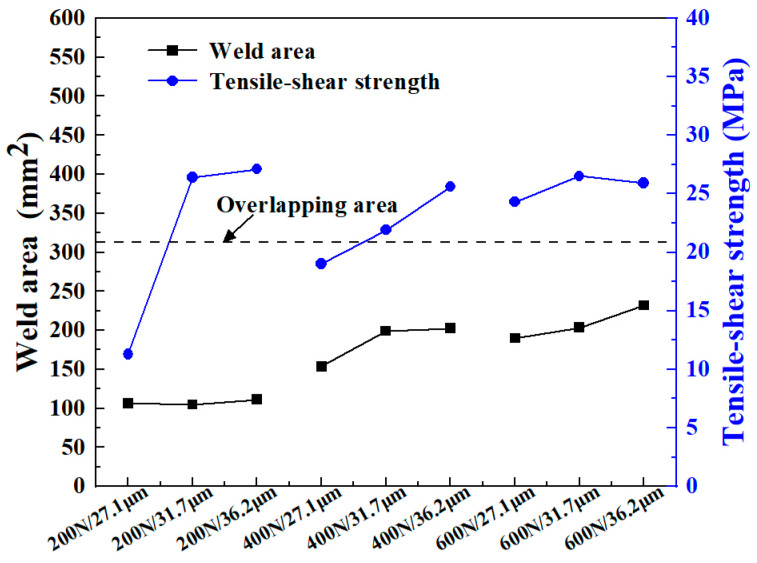
The relationship of weld area and joint strength of the CF/PA6 composite for different welding parameters.

Figure 13b shows the joint failure load. It seems that the failure load is proportional to the weld area. However, the weld quality is not solely contingent on the quantity of the molten material at the welding interface, but also on the intrinsic properties of the weld material. Figure 14 quantifies the weld area and joint strength of the CF/PA6 for different welding parameters. If the joint strength is only related to the weld area, then the blue lines (joint strength) should be parallel to the black lines (weld area). When the weld area is small, other factors such as DoC and stress state could have a large and positive impact on the joint strength. At this time, the slope of the blue lines is much greater than that of the black lines. As the weld area increased, the influence of the welding area on the joint strength gradually increased, which is manifested in the gradual decrease of the slope of the blue lines. However, it is difficult to judge the impact of the interface DoC on the mechanical property of joints simply through experiments only. More in-depth research needs to be carried out with the help of mathematic models or numerical simulations, which will be conducted in future work.

## 4. Conclusions

This study investigated the effect of the pre-weld heat treatment and welding parameters on the ultrasonically welded joint performance from the perspective of DoC. The main conclusions can be drawn as follows.

(1) The pre-weld heat treatment promoted the crystallization of the CF/PA6 base material and the DoC increased slightly with increasing heat-treatment temperature. The maximum DoC (33%) was obtained under the heat-treatment process of 180-O.

(2) Heat treatment could be used to modify the α/γ ratio of the CF/PA6. At temperatures of 100 and 140 °C, the transformation of the amorphous phase into the α form was predominantly promoted, whereas at 180 °C, the transformation of the γ form into the α form was also facilitated.

(3) The increase in DoC and α/γ ratio obtained by heat treatment increased the modulus and UTS of the CF/PA6 base material, resulting in a more efficient USW process and better weld performance. The joint strength of 180-O sheets reached 17.68 MPa, which is 26.7% higher than that of the as-received sheets.

(4) Temperature measurements revealed that higher welding force and amplitude increased the cooling rate at the welding interface, with the cooling rates of HF-HA being 232 °C/s and 58.43 °C/s in the ranges of 100~150 °C and 150~220 °C, respectively. Since the critical cooling rate for PA6 crystallization was 100 °C/s, the crystallization of PA6 during the cooling process occurred mainly between 100 °C and 150 °C.

(5) The DoC at the welding interface decreased with decreasing welding force and vibration, which was inconsistent with the relationship between cooling rates and the DoC. The markedly high strain rates associated with ultrasonic welding are believed to induce PA6 crystallization. It facilitated crystal formation by orienting the molecular chains under high strain rates.

(6) The single-lap shear tests demonstrated a consistent pattern of increasing failure load with DoC at the welding interface. The relationship between joint strength and weld area validated the fact that the joint strength was not only related to the amount of material melted, but to the DoC and other potential factors at the welding interface. However, it is challenging to ascertain the effect of interfacial DoC on the mechanical properties of the joints solely through experiments. A more in-depth study, utilizing mathematical and physical models or multi-scale simulation, is required, and this will be conducted in the future.

## Figures and Tables

**Table 1:** Welding-process parameters for the first part of experiments.

Welding Parameters	Value
Welding energy (J)	500/600/700
Vibration amplitude (μm)	27.1/31.7/36.2
Welding force (N)	200/400/600
Trigger force (N)	600

**Table 2:** Welding parameters for the second part of experiments.

Specimens	Welding Force (N)	Vibration Amplitude (μm)	Welding Energy (J)
Low Force and Low Amplitude (LF-LA)	200	27.1	600
Medium Force and Medium Amplitude (MF-MA)	400	31.7	600
High Force and High Amplitude (HF-HA)	600	36.2	600

**Table 3:** Heat-treatment process.

Heat Treatment	Heat-Treatment Temperature (°C)	Cooling Method
100-O	100	cooling with heated oven
100-A	100	cooling in air
100-C	100	cooling by copper block
140-O	140	cooling with heated oven
140-A	140	cooling in air
140-C	140	cooling by copper block
180-O	180	cooling with heated oven
180-A	180	cooling in air
180-C	180	cooling by copper block

**Table 4:** Structural parameters of α and γ forms of PA6 [18,26].

Property	α Form	γ Form
Crystal structure	Monoclinic	Hexagonal/pseudohexagonal
Lattice constants	a = 9.587 Ả	a = 4.931 Ả
	b = 17.602 Ả	b = 17.267 Ả
	c = 7.760 Ả	c = 8.810 Ả
	β = 69°	β = 126.8°
Melting point (°C)	215	272
Density (g/cm^3^)	1.232	1.16
$\mathrm{Heat}\;\mathrm{of}\;\mathrm{fusion},\;\Delta H_{m}^{o}$ (J/g)	241	239
Young’s modulus (GPa)	235.29	131.97

**Table 5:** The specific peak temperatures of different heat treatments.

Heat Treatment	100-O	100-A	100-C	140-O	140-A	140-C	180-O	180-A	180-C
T_m1_ (°C)	123.9	121.9	120.9	152.9	146	149.9	191	188.1	192.4
T_m2_ (°C)	220.9	219.9	219.8	221.1	220	221	220	221.1	220.3

**Table 6:** Maximum power and vibration time for different combinations of welding force and amplitude.

Welding Force and Vibration Amplitude	Maximum Power (W) (Cov %)	Vibration Time (ms) (Cov %)
200 N/27.1 μm	473 ± 61 (12.9)	1667 ± 187 (11.2)
400 N/27.1 μm	848 ± 58 (6.9)	1147 ± 73 (6.4)
600 N/27.1 μm	1248 ± 35 (2.8)	794 ± 124 (15.7)
200 N/31.7 μm	567 ± 23 (4.0)	1279 ± 53 (4.1)
400 N/31.7 μm	1001 ± 80 (8.0)	790 ± 20 (2.5)
600 N/31.7 μm	1577 ± 148 (9.4)	626 ± 82 (13.1)
200 N/36.2 μm	726 ± 34 (4.7)	1069 ± 88 (8.3)
400 N/36.2 μm	1355 ± 54 (4.0)	683 ± 15 (2.1)
600 N/36.2 μm	2061 ± 137 (6.6)	440 ± 24 (5.5)

**Table 7:** The cooling rates in smaller regions of HF-HA, MF-MA and LF-LA.

Temperature Region	220~150 °C	150~100 °C
HF-HA	232 °C/s	58.4 °C/s
MF-MA	136.4 °C/s	49.1 °C/s
LF-LA	96.4 °C/s	18.4 °C/s

**Table 8:** Parameters of crystallization behavior obtained from DSC tests.

Specimen	ΔHm (J/g)	DoC (%) (CoV %)	Tm (°C)	Tc (°C)	ΔHcc (J/g)
ED_ref	74.48 ± 6.29	31.1 ± 2.62 (8.4)	221.65 ± 1.39	--	--
ED_HF-HA	62.14 ± 5.09	22.9 ± 2.12 (10)	222.87 ± 0.79	108.01 ± 0.57	6.99 ± 1.39
ED_MF-MA	53.96 ± 10.32	19.8 ± 4.34 (21.9)	222.00 ± 0.36	111.32 ± 2.18	4.87 ± 1.49
ED_LF-LA	51.07 ± 7.92	18.3 ± 3.37 (18.4)	223.14 ± 0.93	112.07 ± 2.93	3.89 ± 1.06

## Data Availability

The original contributions presented in this study are included in the article. Further inquiries can be directed to the corresponding author.
